# The Cole Relaxation Frequency as a Parameter to Identify Cancer in Lung Tissue: Preliminary Animal and Ex Vivo Patient Studies

**DOI:** 10.2196/35346

**Published:** 2022-02-21

**Authors:** Les Bogdanowicz, Onur Fidaner, Donato Ceres, Alexander Grycuk, Martina Guidetti, David Demos

**Affiliations:** 1 Novascan Inc Chicago, IL United States; 2 Department of Biomedical Engineering University of Illinois at Chicago Chicago, IL United States; 3 Aurora St. Luke’s Medical Center Milwaukee, WI United States; 4 Department of Cardiothoracic Surgery Aurora Healthcare Milwaukee, WI United States

**Keywords:** Cancer, Detection, Lung Cancer, Non Small Cell Lung Cancer, Spectral Impedance, Cole Relaxation Frequency, biomedical engineering, medical device, scan, testing, identify, lungs, respiratory, diagnosis, model, animal testing, in-vivo, human testing

## Abstract

**Background:**

Lung cancer is the world’s leading cause of cancer deaths, and diagnosis remains challenging. Lung cancer starts as small nodules; early and accurate diagnosis allows timely surgical resection of malignant nodules while avoiding unnecessary surgery in patients with benign nodules.

**Objective:**

The Cole relaxation frequency (CRF) is a derived electrical bioimpedance signature, which may be utilized to distinguish cancerous tissues from normal tissues.

**Methods:**

Human testing ex vivo was conducted with NoduleScan in freshly resected lung tissue from 30 volunteer patients undergoing resection for nonsmall cell lung cancer. The CRF of the tumor and the distant normal lung tissue relative to the tumor were compared to histopathology specimens to establish a potential algorithm for point-of-care diagnosis. For animal testing in vivo, 20 mice were implanted with xenograft human lung cancer tumor cells injected subcutaneously into the right flank of each mouse. Spectral impedance measurements were taken on the tumors on live animals transcutaneously and on the tumors after euthanasia. These CRF measurements were compared to healthy mouse lung tissue. For porcine lung testing ex vivo, porcine lungs were received with the trachea. After removal of the vocal box, a ventilator was attached to pressurize the lung and simulate breathing. At different locations of the lobes, the lung's surface was cut to produce a pocket that could accommodate tumors obtained from in vivo animal testing. The tumors were placed in the subsurface of the lung, and the electrode was placed on top of the lung surface directly over the tumor but with lung tissue between the tumor and the electrode. Spectral impedance measurements were taken when the lungs were in the deflated state, inflated state, and also during the inflation-deflation process to simulate breathing.

**Results:**

Among 60 specimens evaluated in 30 patients, NoduleScan allowed ready discrimination in patients with clear separation of CRF in tumor and distant normal tissue with a high degree of sensitivity (97%) and specificity (87%). In the 25 xenograft small animal model specimens measured, the CRF aligns with the separation observed in the human in vivo measurements. The CRF was successfully measured of tumors implanted into ex vivo porcine lungs, and CRF measurements aligned with previous tests for pressurized and unpressurized lungs.

**Conclusions:**

As previously shown in breast tissue, CRF in the range of 1kHz-10MHz was able to distinguish nonsmall cell lung cancer versus normal tissue. Further, as evidenced by in vivo small animal studies, perfused tumors have the same CRF signature as shown in breast tissue and human ex vivo testing. Inflation and deflation of the lung have no effect on the CRF signature. With additional development, CRF derived from spectral impedance measurements may permit point-of-care diagnosis guiding surgical resection.

## Introduction

### Background

Lung cancer is the cause of 20% of all cancer-related deaths globally and is the most commonly diagnosed cancer worldwide, representing 13% of all cancer diagnoses. Globally it is estimated that lung cancer accounts for 2 million cases per year and 1.7 million deaths per year [1]. The economic health burden of lung cancer is estimated to be 1.5% of the world's gross domestic product, approximately 1.2 trillion dollars [2].

The diagnosis of lung cancer begins with the detection of pulmonary nodules or masses using chest X-rays and low-dose computed tomography (CT) technologies. Nodules and/or masses appear in 1 out of every 500 chest X-rays, and only about 40% are cancerous [3]. These nodules usually develop in the deep periphery of the lung, requiring navigation to branching airway structures to reach them. The nonsurgical gold standard technique to differentiate benign from malignant pulmonary nodules is either a transthoracic needle biopsy or bronchoscopy biopsies [4]. These techniques report a diagnostic accuracy below 78% and 60%, respectively, with decreasing sensitivity for smaller nodules [4,5]. Consequently, when small nodules (<1 cm) are detected, they are typically monitored for 3 to 6 months and are pursued upon growth. More advanced technologies that can detect cancer accurately in small malignant lung nodules can help achieve early diagnosis and prompt treatment, which is critical to lung cancer survival. In recent years low-dose CT screening was shown to reduce lung cancer mortality by shifting cancer detection from symptomatic late-stage cancers to early-stage cancers, supported by the National Lung Screening Trial and the NELSON trial [6]. The relative survival rate for cancerous nodules is about 50% after 5 years, rising to 80% if the nodule is first diagnosed at a diameter of 1 cm or less [3]. In this paper, the authors propose a novel technology, NoduleScan, that provides an accurate diagnosis of cancerous lung nodules via an electrical assessment of the tissue. The accuracy of NoduleScan will be tested by comparing the outcomes (cancerous vs noncancerous) obtained with the new technology to the results of the pathology report on 3 different tissues: (1) human lung tumors from 30 patients (human lung testing ex vivo); (2) A549 human lung tumor xenograft grown on mouse model and noncancerous lung tissue from the same mouse model, tested both in vivo, to include the effect of perfusion, and ex vivo (animal testing); and (3) healthy ventilated porcine lungs with A549 cancerous nodules insertion to include the effect of breathing and to demonstrate detection sensitivity (porcine lung testing). We hypothesized that NoduleScan would discern between cancerous and noncancerous, as was previously shown in breast and skin [7-9].

### NoduleScan Technology

#### Bioimpedance Modeling for Cancer Detection

**Figure 1:**
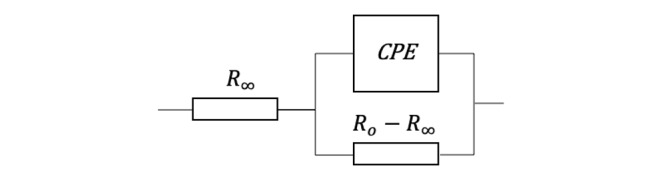
Equivalent circuit model. CPE: constant phase element.

The characterization of the bioelectrical properties of tissues is often achieved using electrical circuits models, which attempt to describe in electrical terms the frequency-dependent behavior of ion movement within the extracellular and intracellular media and across the cellular membrane, as well as membrane polarization effects. The Cole-Cole impedance model (Figure 1) is often used to describe the impedance behavior of biological tissue [10].

In this representation, the constant phase element (CPE) is an empirical circuit element purely devised to describe polarization phenomena as a result of system inhomogeneity:



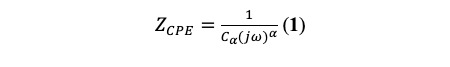



where *C_α_* is a pseudo-capacitance, and α is its order (0<α<1).

The Cole-Cole equation (2) derives in principle from the CPE model, specifically when describing impedance behavior of biological tissue as



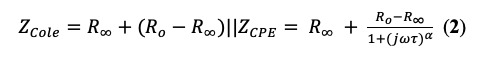



Where *τ=1/2πf_c_* is the relaxation constant, *C_α_=τ^α^/(R_0_-R_∞_),* and *f_c_* is the Cole relaxation frequency (CRF) [11].

The fundamental parameters (*R_0_*, *R_∞_*, and *f_c_*) can be used to describe and characterize the bioimpedance behavior of a tissue. Although these parameters do not necessarily describe the physical mechanisms involved in the impedance measurement, in the literature, *R_0_* and *R_∞_* have been associated with the extracellular medium and fluids, while α and *C_α_* with membrane polarization and ionic permeability [12-14]. Accordingly, we have found that only the CRF might be sufficient to discriminate between cancerous and noncancerous tissue in the frequency range from 10^3^ Hz to 10^7^ Hz, often referred to as the β dispersion region, where cellular membranes have the strongest frequency response [8]. This enhanced dispersion sensitivity of the cellular membrane in the β region might provide the physiological interpretation of why the differential membrane polarization and ionic permeability between healthy and cancerous cells in a given tissue result in distinct impedance signatures, and more particularly of CRF values [15].

Recently, it has been demonstrated in a cohort of 373 patients that a single parameter, CRF, can be used to detect breast cancer with sensitivity and specificity of 95% and 87%, respectively [8]. Similarly, CRF has been used to identify skin cancer with sensitivity and specificity of 100% and 90%, respectively [7,9]. In the same way, the CFR parameter will be used in the present study as a biomarker for lung cancer detection, based on impedance spectroscopy in the β frequency range [15].

#### NoduleScan Device Used for Testing

**Figure 2:**
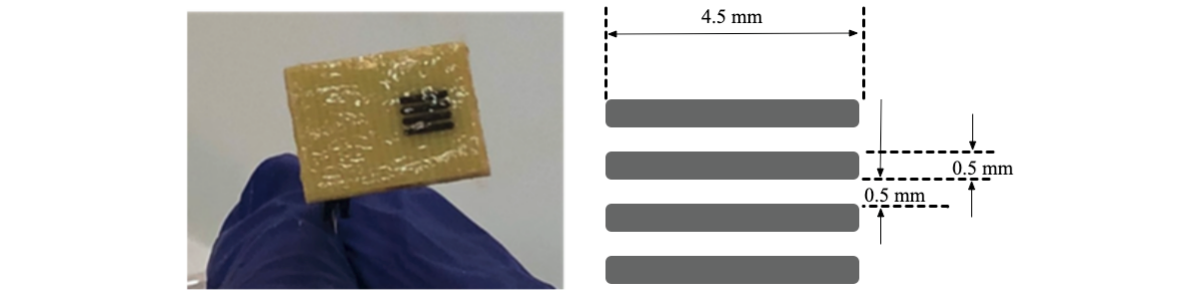
Photograph and dimensions of measuring electrodes.

The CRF measurements are obtained using a tetrapolar electrode system, as shown in Figure 2. The 4 electrodes are prepared by electroplating platinum black (PtBlk), which results in nonpolarizable electrode surfaces suitable for bioimpedance measurement of tissue. An electrolytic gel [16] is used to provide an electrical coupling between the electrodes and the tissue. When in contact with the tissue, a spectral impedance measurement is recorded at discrete frequencies (32 frequencies per decade) in the β frequency region. In this tetrapolar configuration, the two outer electrodes are the stimulating electrodes that drive a current through the tissue by applying a 1V_rms_ voltage, while the two inner electrodes are used to measure the voltage differential. Amplitude and phase of complex bioelectrical impedance are then calculated from the ratio of the voltage differential to the current. The impedance measurement is repeated for each discrete frequency in the β frequency region. Our testing apparatus is optimized to have the highest fidelity in the 2x10^3^Hz-1x10^7^ Hz frequency range.

#### Analysis of Impedance Data

The raw impedance data is analyzed through the following steps.

1. Remove parasitic resistance-capacitance artifacts coming from the reactive circuit elements in the probe and the cable.
2. Nyquist path analysis of data for initial determination of cancer
3. Find α and f_c_ values that result in the highest correlation between the impedance formula in equation (1) and the data.
  - If the CRF value (f_c_) is in the range of 10^5^ Hz-2.1 x 10^6^ Hz (defined as the cancerous range) [8], then the scan is characterized as “cancer,” otherwise “no cancer.”
  - If there is no CRF value (f_c_) present, then the scan is characterized as “no cancer,” solely based on Nyquist path analysis results.
4. Typically, multiple scans are taken from each sample at slightly different locations. If at least one scan is cancer, then the sample is characterized as “cancer.” Otherwise, if all scans from a sample are “no cancer,” then the sample is characterized as “no cancer.”

**Figure 3:**
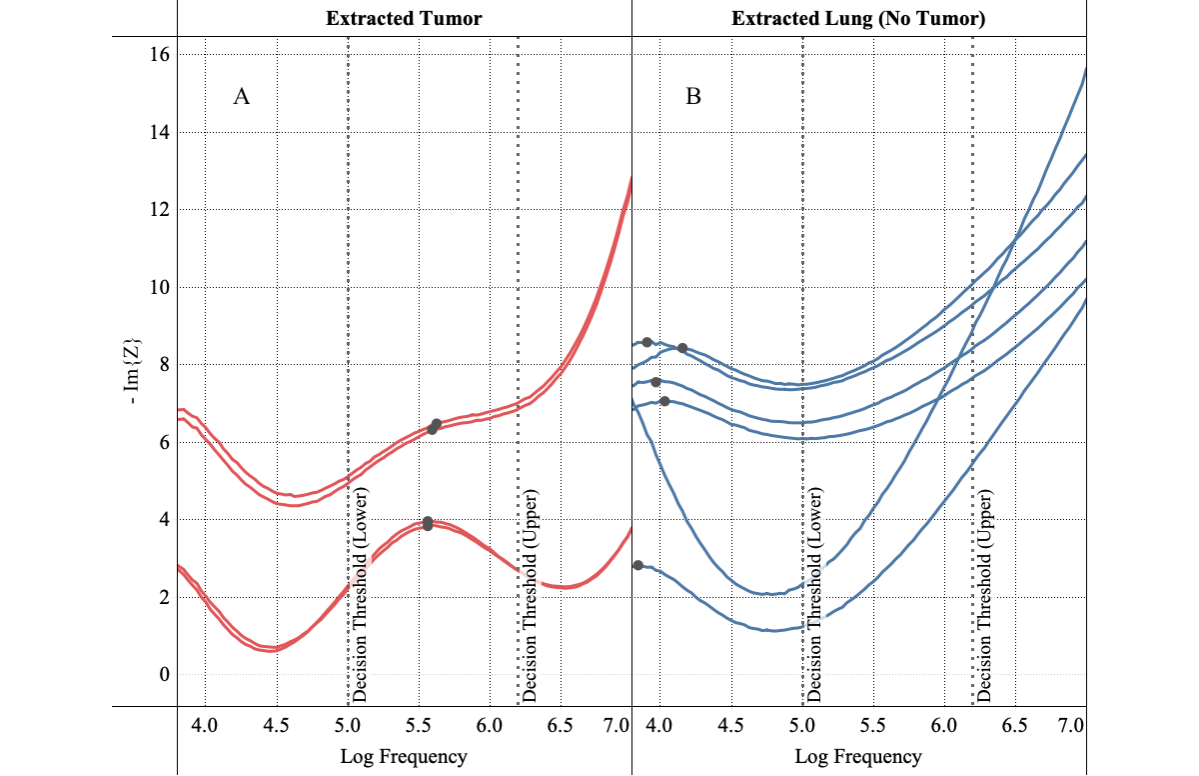
Sample of spectral impedance measurements obtained from mouse model tests. Multiple curves represent repeated measurements of the same tissue in slightly different locations. (a) A549 tumor extracted from mouse (b) normal lung tissue extracted from mouse.

**Figure 4:**
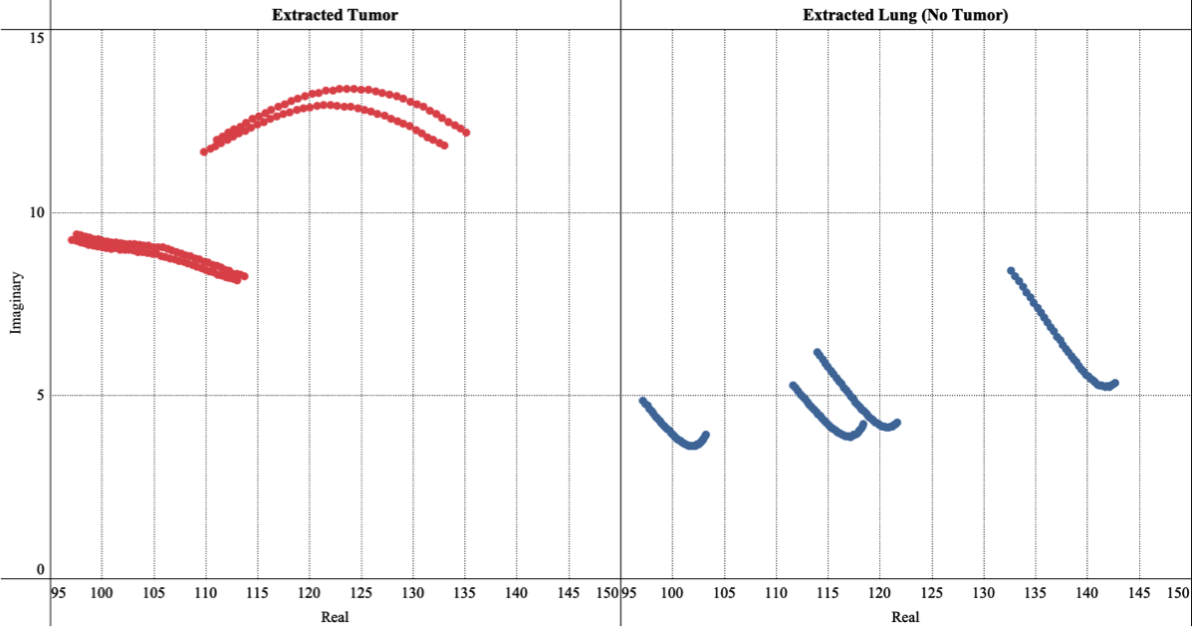
Nyquist path plot, which shows the imaginary part of measured impedance vs the real part for data obtained from mouse model tests, for frequencies in the cancer range 1x10^5^ Hz - 2.1x10^5^ Hz.

As an example of the application of the NoduleScan technology, Figure 3 shows the imaginary component of complex impedance computed for two specimens obtained from a mouse model. The plots show the imaginary component of measured impedance following removal of parasitic capacitance components. Figure 3A shows the impedance response of an A549 tumor grown on a mouse, which was confirmed to be “cancer” via pathology. For this sample, our scans detected clear peaks at about 6x10^5^ Hz, categorizing it as “cancer.” Figure 3B shows the impedance response of a healthy lung sample extracted from the same mouse, which was confirmed to be “no cancer” via pathology. For this sample, no peaks could be detected in the cancer range; hence the sample was categorized as “no cancer.” Figure 4 shows the Nyquist path plots of the same data set in Figure 3 for data points in the cancer frequency range of 1x10^5^-2.1x10^6^ Hz. Cancer scans tend to have a concave arch shape, as suggested by the Cole function (Eq.1 and is the basis for initial cancer/no cancer classification in Step 2.

## Methods

### Human Lung Testing Ex Vivo

Patients (n=30) were recruited by informed consent from the partner Aurora St. Luke’s Medical Center in Milwaukee, Wisconsin, under the approval from WCG IRB (study number: 1264221). Research subjects were recruited from patients at any site within the Aurora Healthcare system, who had a diagnosis of nonsmall cell lung carcinoma (NSCLC) with tumor size >1.4 cm, and were elected for a surgical wedge segmentectomy or lobectomy procedure with or without lymph node dissection.

- *Study population:* Adult men and women presenting with a diagnosis of NSCLC who were candidates for wedge resection or anatomic resection, or thoracic nodal dissection.
- *Inclusion criteria:* Adult male and female patients, 18 years of age or greater, with a biopsy-proven diagnosis of lung cancer and who are scheduled for a wedge resection or anatomic resection. Preoperative diagnosis is NSCLC that may include the following: invasive carcinoma, squamous cell carcinoma, or adenocarcinoma for those patients scheduled for surgical excision of tissue.
- *Exclusion criteria:* Patients who refuse consent for the study of their excised tissue, patients with a diagnosis of small cell lung cancer, patients with a diagnosis of NSCLC who underwent induction therapy with either chemotherapy or radiation, and patients who are not candidates for surgical resection.

Research subjects were preoperatively consented to by the surgeon and Aurora research coordinator. The patient then underwent a lobectomy or segmentectomy as per standard of care, and the excisions were bisected. The authors collaborated with the pathology staff to select tissue from two sites for each patient: a piece from the suspected cancerous region and a piece from noncancerous tissue at least 5 cm away from the suspected tumor site. CRF measurements were performed by placing the electrode in different positions on each tissue sample (tumor and away-from-tumor). Once spectral impedance scanning was completed (multiple scans for both tumor and away-from-tumor pieces), the tissue samples were returned to the pathology staff for immediate processing and histological evaluation.

### Animal Testing

#### Overview

With encouraging results from the human ex-vivo lung assessment, we pursued answering questions on the ability to discern between cancer and noncancer in perfused tissue. Twenty mice were inoculated with an NSCLC line, and all survived to full tumor growth. CRF measurements were taken on tumors through the skin with a live animal, with the tumor exposed after euthanizing the animal, and subsequently, the tumor itself was excised and measured. These results were compared to measurements on the excised healthy lung tissue from the same mouse.

Bagg Albino inbred nude mice BALB/c NU/NU (n=25) were implanted with tumor cells that were grown in culture (A549 lung sarcoma cells obtained from American Type Culture Collection, ATCC: CCL-185). These A549 cells (2 × 106 cells in approximately 100 µL sterile PBS) were injected subcutaneously into the right flank of each mouse.

Animals with tumor sizes between 0.1 cm^3^ to 1.0 cm^3^ were utilized in this study. Tumor measurements were monitored twice weekly until termination. Tumor size was calculated using the formula: volume=½ Length x width^2^.

Animal testing was performed at the University of Illinois (UIC) Toxicology Research Laboratory (TRL). The protocol for the study was approved by the UIC Office of Animal Care and Institutional Biosafety (approval number: 20-147).

#### Spectral Impedance Measurements

1. *Impedance measurements on tumors with skin intact on live mice:* CRF was measured on live mice on the surface of the intact skin at the tumor site. The live mice were held steady by the nape of their neck and tail, and measurements were taken on the tumor site holding the electrodes in place. Upon completion of this test, the mice were euthanized.
2. *Impedance measurements on dead mice with exposed tumor:* The tumor site was exposed by cutting a flap from the posterior end of the mouse, extending anteriorly until the entirety of the tumor was accessible. The measurement electrode was then placed directly on the exposed tumor, and multiple spectral impedance scans were recorded.
3. *Impedance measurements on ex vivo tumor:* The tumor was excised in its entirety from the animal and bisected. The sliced faces of the tumor pieces were then placed on the measurement electrode one by one, and spectral impedance scans were taken from each piece. One piece was sent to histology for evaluation.
4. *Impedance measurements on noncancerous excised mice lung:* The lungs (noncancerous tissue) were excised from the animal and sliced so that the interior faces were exposed. Spectral impedance measurement scans were recorded. One piece was sent to histology for evaluation.

### Porcine Lung Testing

#### Overview

Two healthy porcine lungs were received within 24 to 26 hours of euthanization with the trachea and vocal cords intact and shipped to the research facility overnight at a constant temperature of 4°C. The vocal cords were cut out, and a manual ventilator was attached and secured tightly in place. The lungs were inflated and deflated using the ventilator, and the applied pressure was measured using a gauge.

Separately, 5 human lung tumors (A549 cell line) were grown in 5 separate mice following the procedure in the murine xenograft cancer method discussed earlier. Following tumor inoculation in mice, tumor size was monitored regularly. Once tumor size exceeded 500 mm^3^, the mouse was euthanized, and the tumor was excised and bisected. Consequently, each bisected piece size was greater than 250 mm^3^ on average and ready to be inserted in the porcine lung tissue in order to undergo spectral measurements during simulated breathing.

#### Spectral Impedance Measurements

1. *Impedance measurements on ex vivo tumor:* Spectral impedance measurements were recorded on A549 tumors immediately after excision and bisection. The measurements were done ex vivo on the inner bisected surface of the tumors. One bisected piece was placed in 10% formalin and sent to a histology laboratory for evaluation.
2. *Impedance measurements on healthy porcine lung tissue:* Spectral impedance measurements were performed on porcine lungs when the lung was deflated. Subsequently, the lung was pressurized to 20 mmHg and kept at an inflated state with constant pressure. Lastly, the air was slowly released to deflate the lung at a rate of <1 mmHg per second. During the deflation process, multiple spectral impedance scans were performed.
3. *Impedance measurements on porcine lung with inserted tumor:* A flap was created on the lung to expose the underlying subsurface lung tissue by cutting the outermost pleural layer of the lung. The flap tissue was cut to a width that matched the size of the A549 tumor previously excised from the mouse in order to ensure the tumor fit in the lung subsurface. The thickness of the flap was measured and recorded using a caliper; the target cut tissue thickness was <300 microns. The bisected A549 tumors were placed in the created lung subsurface with the flap covering the tumor. Measurements were taken on the surface of the lung at the tumor location, with the lungs in an inflated and deflated state. This was repeated for each of the tumor samples. Saline solution was applied to the lungs every 15 min during the studies to maintain tissue hydration.

## Results

### Human Lung Testing Outcomes

Patients’ demographic and tumor characteristics are presented in Table 1.

**Figure 5:**
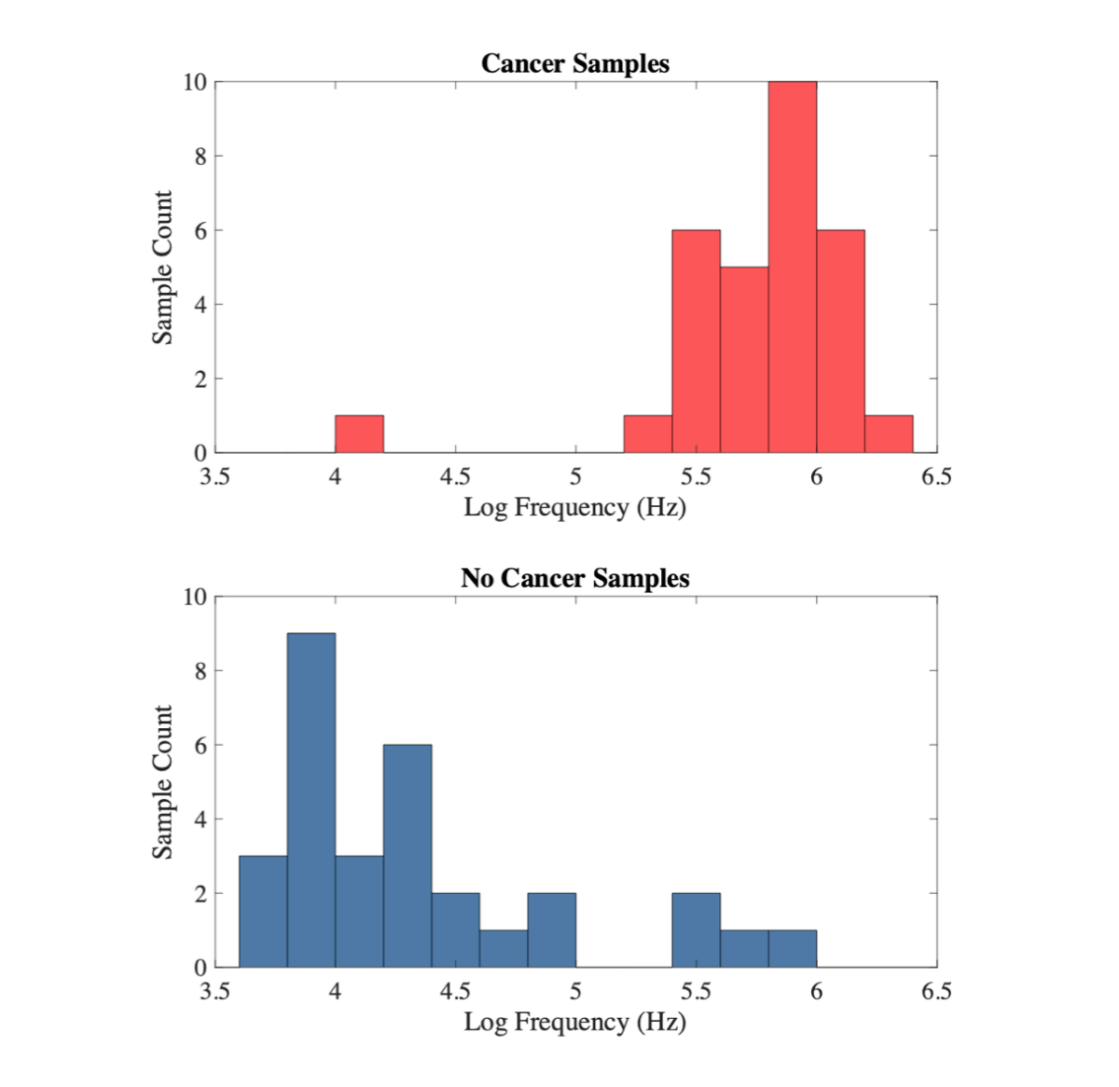
Histogram of Cole relaxation frequency (CRF) measurements from human lung samples.

The maximum tumor size presented was 7.5 cm. Per the protocol, subjects with tumor sizes larger than 1.4 cm were included in the study. The mean size of the tumors was 3.22 cm and median 3.0 cm, with a standard deviation of 1.47 cm. Figure 5 top plot shows a histogram of CRF values cancerous tumor samples, as determined by pathology. The bottom plot shows the CRF measurements determined to be healthy tissue by the pathologist. Note that multiple scans were taken per sample. When cancer scans are present, a sample is categorized as “cancer,” and below threshold scans are ignored. The data points in the histogram represent the log-mean of measured CRF values.

Cancerous regions for CRF values (1x10^5^ Hz-2.1x10^6^ Hz) were compared to the pathology report for cancerous tissue. One tumor sample (confirmed to be “cancer” via pathology) did not yield a CRF value in the cancerous frequency range, resulting in a false negative. We found CRF values in the cancerous range for three ‘normal’ tissue specimens (confirmed to be “no cancer” via pathology) that were at least 5 cm away from the tumor lesion, resulting in false positives. The overall sensitivity and specificity values (combined tumor and distant tumor tissue) are 97% and 87%, respectively. The positive predictive value is 88%, and the negative predictive value is 96%. Results are summarized in Table 2.

**Table 1:** Patients’ demographic and tumor characteristics.

Characteristics	No. of patients
Patient age group (54-85 years)	30
Males	16
Females	14
Adenocarcinoma	21
Squamous Cell Carcinoma	9
pN0	27
pN2	3
ypT0	1
pT1a	0
pT1b	7
pT1c	7
mpT1c	1
pT2	1
pT2a	4
mpT2a	1
pT2b	3
pT3	3
pT4	2

**Table 2:** Sensitivity and specificity of human lung testing.

		Pathology outcome tumor tissue		Pathology outcome distant tissue (normal)		Overall	
		Cancer	No cancer	Cancer	No cancer	Cancer	No cancer
**CRF^a^ DX^b^**							
	Cancer	28	0	1	4	29	4
	No Cancer	1	1	0	25	1	26

^a^CRF: Cole relaxation frequency.

^b^DX: diagnosis.

### Mouse Model Testing

**Figure 6:**
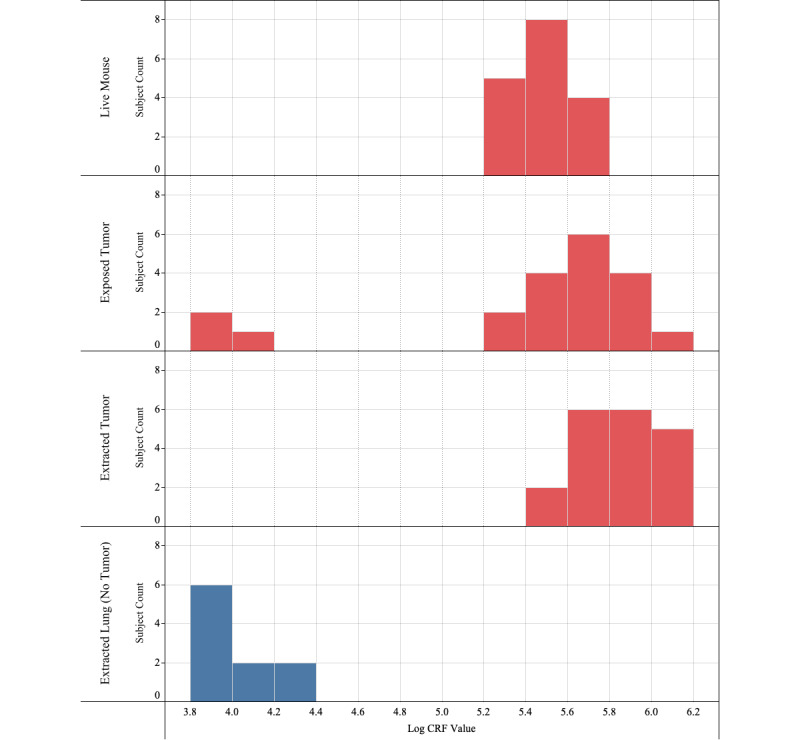
Histogram of Cole relaxation frequency (CRF) measurements of A549 tumors grown on mice.

- *Tumor growth:* Tumor height and diameter were measured for each mouse in regular time intervals following inoculation. The eventual tumor sizes had a mean of 432 mm^3^ and a standard deviation of 222 mm^3^.
- *Impedance measurements on tumors with skin intact on live mice:* CRF peaks were detected in the cancer range for all samples confirmed as tumors from the pathology report. The median CRF value was determined to be 3.11x10^5^ Hz. In some samples, multiple distinct CRF peaks can be observed—a phenomenon reported earlier in the literature [8]. In tumor samples, we typically observed a primary CRF in the higher frequency range, the cancerous range, and a secondary CRF value in the lower frequency range. These secondary CRF values correspond to noncancerous tissue. The presence of noncancerous CRF measurements and cancerous CRF measurements reflect the heterogeneity of lung cancer [17].
- *Impedance measurements on dead mice with exposed tumor:* Impedance measurements were recorded on various spots on the exposed tumor. The median CRF value was determined to be 4.36x10^5^ Hz.
- *Impedance measurements on extracted tumor:* Measurements of the sliced tumor were similar to those of the exposed tumor. The median CRF value of sliced tumors was determined to be 8.32x10^5^ Hz. All histology data confirmed that the tissue pieces were cancerous. This offers a direct comparison of the CRF curves for tumor and no-tumor cases. A comparison of CRF frequency histograms for tumor slices and lung slices is provided in Figure 6 below. The horizontal axis represents the base 10 logarithms of frequency (Hz) values. The histograms are formed using CRF values from live mice, exposed tumor, extracted tumor, and extracted lung (healthy tissue). Typically, 3-4 scans are taken from each sample. When cancer scans are present, a sample is categorized as “cancer,” and below threshold scans are ignored. The data points in the histogram represent the log-mean of measured CRF values.

The analysis reveals that all the CRF measurements for lung slices (no-tumor sample) are below the decision threshold of 1x10^5^ Hz, with a median CRF frequency of 9.68x10^3^ Hz. On the other hand, most of the CRF peaks for tumor slices samples are above the decision threshold, with a median CRF frequency of 8.28x10^5^ Hz.

In Table 3, we show the comparison of pathologist outcome of tumor samples compared to CRF scans and healthy lung tissue compared to CRF measurements. For the 10 samples analyzed, the outcomes corresponded to 100% sensitivity, 100% specificity, and 100% positive predictive value and negative predictive value.

**Table 3:** Sensitivity and specificity of mouse model testing.

		Pathology outcome tumor tissue		Normal lung tissue (no cancer)		Pathology outcome overall	
		Cancer	No cancer	Cancer	No cancer	Cancer	No cancer
**CRF^a^ DX^b^**							
	Cancer	10	0	0	0	10	0
	No cancer	0	0	0	10	0	10

^a^CRF: Cole relaxation frequency.

^b^DX: diagnosis.

### Porcine Lung Testing

#### Impact of Simulated Breathing on CRF Values

**Figure 7:**
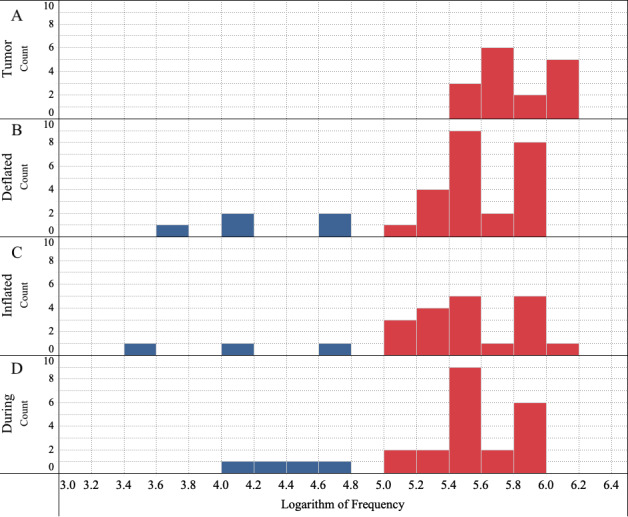
Histograms of Cole relaxation frequency (CRF) data for (A) Tumor Only, (B) Deflated, (C) Inflated, and (D) During Simulated Breathing cases.

To understand the impact of simulated breathing, in Figure 7, we compare the histograms for deflated, inflated, and during simulated breathing cases. Data include subsurface (electrode on flap) and subsurface (electrode on tumor) cases combined. A no CRF peak measured is classified as noncancer. There is a shift in the log-mean of CRF peak frequencies among simulated breathing scenarios (deflated/inflated/during) and is within 0.05 log cycles. There is a smaller amount of CRF measurements in the no cancer range resulting in limited information in histogram plots.

## Discussion

### Principal Findings

We set out to test a novel technology, NoduleScan, to provide a diagnosis of cancerous lung nodules via an electrical assessment of the tissue. Further, we have shown that the diagnosis of cancerous and noncancerous tissue is in line with those seen in breast tissue and skin tissue [7-9].

We have shown that ex vivo CRF measurements of excised human lung cancerous nodules can differentiate cancerous and noncancerous tumors with a sensitivity of 97% and specificity of 87%.

In xenograft mouse models, tumor growth was found to be in agreement with published literature on mice inoculated with the same amount of A549 cells [18]. A549 lung sarcoma tumors grown in mice exhibit the same distinction in CRF values as ex vivo lung tissues and those of previously reported breast and skin tissue. The measurements remain the same when measured in vivo through the skin, with the skin opened tumor exposed and tumor excised. There was no difference in CRF measurements with the tumor perfused or not perfused. When the tumor measurements, confirmed to be cancerous by a pathological examination, were compared to the healthy lung tissue of the sacrificed mouse, the sensitivity and specificity were 100%.

Clear separation of CRF distributions, as shown in Figure 6, demonstrates that CRF is a viable parameter to identify the presence of cancer. Cancer determination is done through multiple scans from the same tissue sample. For each of these below-threshold primary peaks, there was at least one other scan from the same tissue that exhibited cancer-range CRF frequency, hence correctly classifying the sample as “cancer.”

CRF values could be detected and remained relatively unchanged during simulated breathing. CRF values are in the same ranges as shown in ex vivo human tumor tests, xenograft mice, and previously demonstrated breast and skin tissues.

### Conclusions

#### Patients of Interest

Table 2 shows a pathological assessment of a suspect lesion as noncancerous and, secondarily, a normal tissue assessment as cancerous. These patients were patient #6036, a 60 year old female undergoing a right upper lobectomy, and study patient #6086, a 72 year old female undergoing a right lower lobectomy. For patient #6036, the nodule presented did not show any CRF values in the cancerous range, and this was later confirmed by pathological examination as fibrotic tissue, TNM status ypT0, pN0, and no cancer was detected showing concordance with the scanned results. Patient #6086 measured CRF values in the cancerous range for tissue distant from the tumor (>5 cm,) and pathological examination confirmed positive margins and thus showed concordance with our measurements.

#### NoduleScan Technology

The NoduleScan assessment is completed rapidly (seconds to make a full scan). This can be completed without expensive or bulky capital equipment. Further, learning to interpret CRF measurements, while the physician does need to interpret, is straightforward and not nearly as subjective as interpreting confocal microscopy measurements, for example.

#### As a Surgical Tool for Assessing Clear Margins

The aim of surgical therapy for the treatment of lung cancer is to excise malignant tumors with clear margins meaning cancer cells are not present at the divided edge of the tissue that was removed [19]. The definition of an adequate margin width is loosely defined as 2 cm or the same as the diameter of the tumor. The tenet that a positive margin (cancer cells present at the cut edge of the excised tissue) results in an increased incidence of cancer recurrence is widely accepted. A “positive” margin is, in a sense, a surrogate for residual tumor, and those patients have been shown to have an increased risk of local recurrence and mortality [19-21].

Pathology evaluation of margins involves sampling the edges of tissue from all cut faces of the excised specimen. It’s impractical for a pathologist to microscopically examine the entire margin in a specimen. The technology presented allows for electrodes to be embedded on a handheld or robotic surgical tool allowing the surgeon to assess the margin quickly and effectively in the operating theater. In circumstances of a positive margin, adjuvant therapy can be implemented to help eliminate residual local disease [20]. Classification of tumors as microscopically positive (R1) versus tumors with margins grossly involved by tumor (R2) could be impacted by such a point-of-care analysis of a specimen [20,22-27].

#### As a Tool for Collecting Efficient Biopsies

Nondiagnostic rates for biopsies in CT-guided needle biopsies are reported to be 27.6%. Nodules presented smaller than 1 cm have a 40% nondiagnostic rate [28]. One out of 4 biopsies is inefficient, exposing the patient to complications and risk. The electrodes in the system demonstrated herein can be miniaturized and can fit within a 2 mm orifice of the bronchoscope or on the surface of a biopsy needle. Being able to have a device that can give an indication of malignancy without having to directly sample the tissue would give this process a much higher yield as some of those nondiagnostic samples could also come with a CRF that demonstrates the nodule is malignant. The electrodes can be spaced to assess the entirety of a nodule allowing to compensate for the heterogeneity of the nodule. The measurement can provide an accurate, real-time answer on the presence of cancer. Lesions that were previously difficult to locate from the airway in real time can be easily located as the technology can visualize macroscopically from the airway and identify both cancerous and benign nodules. This provides a precise periprocedural intralesional assessment mechanism to confirm the location of cancerous cells within a lesion.

#### Lymph Node Assessment

Lymph node status can often influence the decision to proceed with surgery first or induction chemotherapy (N2 nodes). Further, an N3 lymph node will influence the decision of whether or not surgical resection is indicated at all. These nodes can be assessed either by mediastinoscopy before a planned resection or during the planned resection but before the resection itself has taken place. Real-time nodal assessment with the elimination of the time and resource-intensive frozen pathologic analytical process and its inherent uncertainties would be invaluable in these circumstances.

#### Residual Tumor

There are situations where a patient receives induction chemotherapy and/or radiation therapy followed by surgical resection. This involves 5% to 10% of lung cancer patients but a much larger proportion of esophageal cancer patients. Sometimes their preoperative induction therapy results in complete pathologic response (CPR), in which no viable tumor is detected via pathologic evaluation. Measuring a CRF in this patient population could potentially significantly impact this assessment. In the setting of a CPR, a positive CRF might put that designation into question and have some implications as to recurrence risk. Similarly, in patients who are treated with definitive chemoradiation therapy who are not surgical candidates and are under post-treatment surveillance, the ability to assess the treatment area with a CRF could potentially lead to earlier detection of recurrence. Furthermore, this could prove valuable if a local endoscopic resection margin is uncertain.

## Abbreviations

CPE: constant phase element
CPR: complete pathologic response
CRF: Cole relaxation frequency
CT: computed tomography
NSCLC: nonsmall cell lung carcinoma
TRL: Toxicology Research Laboratory
UIC: University of Illinois
